# Film Blowing of Linear and Long-Chain Branched Poly(ethylene terephthalate)

**DOI:** 10.3390/polym12071605

**Published:** 2020-07-19

**Authors:** Michael Härth, Andrea Dörnhöfer

**Affiliations:** 1 Institute of Polymer Materials, Friedrich-Alexander-University Erlangen-Nuremberg, Martensstraße 7, 91058 Erlangen, Germany; adoernho@wlgore.com; 2 Kuraray Europe GmbH, Mülheimer Str. 26, 53840 Troisdorf, Germany; 3 W. L. Gore & Associates GmbH, Hermann-Oberth-Str. 22, 85640 Putzbrunn, Germany

**Keywords:** poly(ethylene terephthalate), chain extender, long-chain branches, rheology, extensional viscosity, film blowing, film uniformity

## Abstract

Film blowing of Poly(ethylene terephthalate) (PET) is challenging due its inherently low melt viscosity and poor melt strength. In this study, it is shown how the rheological properties of a commercial PET can be altered by reactive extrusion using either pyromellitic dianhydride (PMDA) or a multifunctional epoxy (Joncryl^®^ ADR 4368) as chain extender, in order to improve the processing behavior during film blowing. The modified materials were characterized by shear and elongation rheometry and relevant processing characteristics, like melt pressure, bubble stability, and film thickness uniformity, were used to assess the influence of the type of modifier on processing and product performance. It is shown that PMDA is useful to increase the melt strength which leads to an improved bubble stability, while epoxy modified PET shows a reduced drawability that can cause problems at high take-up ratios. On the other hand, the epoxy modifier indicates a pronounced strain hardening during elongational deformation, and therefore leads to a better film thickness uniformity compared to the neat PET and the PET modified with PMDA. The differences with respect to processing performance are discussed and ascribed to the molecular structure of the materials.

## 1. Introduction

Polyester films used, e.g., for packaging, adhesive tapes, medical applications, or furniture surfaces, are generally produced by the cast-film technique with a subsequent biaxial stretching process. The biaxial stretching takes place in the temperature range between the glass-transition and the cold-crystallization temperature and improves end-use properties, like tensile strength or gas permeability. Typical film thicknesses of commercially available biaxially-oriented Poly(ethylene terephthalate) (BO-PET) are between six and 500 µm [1].

PET resins are historically classified by their intrinsic viscosity [η] and are selected based on the manufacturing process requirements and the end-use application. For BO-PET, [η] values between 0.6 and 0.7 dL/g are common [2]. At relevant processing temperatures, the shear viscosity curves of such resins show a pronounced Newtonian plateau and a relatively low zero shear viscosity η_0_ compared to other polymers (e.g., [3,4,5]). This viscosity behavior is beneficial for the cast-film process and can be ascribed to a low weight average molar mass M_w_ and a linear molecule structure. One the other hand, these special molecular characteristics lead to a low melt strength which is of disadvantage for other processes, like blow molding or film blowing. To overcome this deficiency high Mw resins ([η] = 0.7–0.85 dL/g [2]) are typically used for PET bottles manufactured by blow molding. Furthermore, special machine equipment can be used in the case of blown PET films [6]. An alternative route to increase the melt strength and to make PET applicable for other processes is to change the molecular architecture. Over the last decades, the reactive extrusion of PET with chain extenders has been proven as a fast and cheap method to increase [η] of, e.g., recycled PET and/or to introduce long-chain branches (e.g., [7,8,9,10,11]).

In this study, we compare two well-known chain extenders (pyromellitic dianhydride (PMDA) and Joncryl^®^ ADR 4368) with respect to their applicability to improve the processing behavior of PET in the film blowing process. Film blowing can be seen as a cheaper alternative to the BO-PET cast-film process [12] by creating less edge scrap as the biaxial stretching can be obtained with a double bubble set-up [2].

In the literature, investigations of chain extended PET for the film blowing process are very rare. Di Maio et al. [13] used recycled PET with [η] = 0.48 dL/g and PMDA as chain extender in concentrations between 0.25 and 0.75 wt.%. While no films could be blown with the neat PET, the authors found an improved bubble stability with increasing PMDA concentration. This has been correlated to an enhanced melt strength and is in line with investigations on polyethylene [14]. Film blowing experiments of PET modified with Joncryl could not be found in the literature. However, investigations with poly(lactic acid) (PLA), reactively processed with Joncryl, reveal a better bubble stability and an enlargement of the processing window compared to the neat PLA [15,16].

As the question concerning which of the two chain extenders is more suitable for film blowing applications is of high practical and industrial relevance, the aim of this contribution is to modify commercial PET with either PMDA or Joncryl and to compare the processing behavior. In particular, the molecular and rheological properties were analyzed and correlated to processing and end-product quantities, like extrusion pressure, bubble stability, and film thickness uniformity.

## 2. Experimental

### 2.1. Materials

A high molar mass PET copolymer (Artenius Care, La Seda de Barcelona) has been used, as it can be film blown without a chain extender, and therefore used as a reference. According to the supplier, the intrinsic viscosity is 1.15 dL/g. The number and weight average molar mass of the as received pellets have been determined by size-exclusion-chromatography (SEC) with triple-detection to M_n_ = 44 kg/mol and M_w_ = 86 kg/mol. PMDA, which is a tetra-functional modifier was purchased from Sigma-Aldrich and Joncryl^®^ ADR 4368, which is an oligomeric multifunctional epoxy modifier, was provided by BASF. More details regarding the properties and molecular structure of the modifiers can be found elsewhere [8,9].

### 2.2. Reactive Extrusion

The PMDA powder and PET pellets were blended in a tumbling mixer and afterwards pre-dried at 130 °C for 24 h. As the Joncryl modifier has a glass transition temperature of 54 °C [17], the PET was dried separately and mixed with the non-dried chain extender directly before performing the reactive extrusion. The mixture of PET pellets and chain extender was fed into the utilized co-rotating twin screw extruder (Leistritz LSM 34 GL, L/D ratio = 32) by gravimetric dosing, which was purged with nitrogen to avoid moisture up-take. The screw rotation speed was set to 30 rpm and the temperature in the feeding zone to 270 °C and in the metering zone to 290 °C, respectively. The modified materials were quenched in a water bath and afterwards granulated.

Based on a comprehensive pre-study, using the same materials, but a kneader for performing the reactive processing [8,9], the PMDA concentrations have been chosen as 0.1 and 0.25 wt.%. These low concentrations already lead to a significant viscosity increase without forming gels. The utilized Joncryl concentration was 0.4 wt.%, as lower concentrations lead to only a minor viscosity increase, whereas at higher concentrations a significant gel formation occurs [9,11].

### 2.3. Molecular and Rheological Characterization

The SEC measurements were performed with a GPCmax (Malvern, PA, USA) with triple detection (TDA 305, Malvern, PA, USA) and potential gel contents were determined by solvent extraction. Details can be found elsewhere [8].

For the rheological characterization in shear, an ARES rheometer (TA Instruments) with plate-plate geometry was used. Contrarily to the pre-investigations to this study [8,9], the materials were not press molded before the rheological characterization in order to reduce thermal degradation. Instead, the pellets were directly loaded into the pre-heated rheometer by using a metal ring. The time-dependent elongational viscosity was measured with the extensional viscosity fixture (EVF, TA Instruments) as described in Härth et al. [8].

The melt strengths of the neat PET and the modified samples were determined with a Rheotens 71.97 (Göttfert). The temperature of the utilized capillary rheometer (Rheograph 2003, Göttfert) was set to 270 °C and the piston speed to 1 mm/s. The diameter and length of the die was 2 mm and 20 mm, respectively. The spinning length was chosen as 100 mm and the acceleration of the Rheotens rolls was 120 mm/s^2^. More details regarding the Rheotens and the experimental procedure can be found elsewhere [18,19].

### 2.4. Film Blowing

The film blowing experiments were conducted with a laboratory device from the Göttfert company. A schematic drawing of the experimental set-up is shown in [14]. The set-up is equipped with a single screw extruder with a screw diameter of 30 mm and a screw length of 600 mm. The temperature was set to 270 °C in the feeding zone and 280 °C in the metering zone. The throughput was kept constant for all materials at 4.4 kg/h and the cooling system was varied in such a way that a constant frost line for all experimental set-ups was obtained. The blow-up ratio (**BUR**), defined as

(1) $$BUR=\frac{d}{d_{0}}$$

with **d** being the diameter of the blown-up bubble, and **d_0_** the diameter of the annular die, which was set to 2, and the take-up ratio (**TUR**), defined as

(2) $$TUR=\frac{v}{v_{0}}$$

with **v** being the velocity of the take-up rolls and **v_0_** the velocity of the melt at the die exit was varied between 8 and 42.

## 3. Results and Discussion

### 3.1. Molecular Characterization

**Figure 1:**
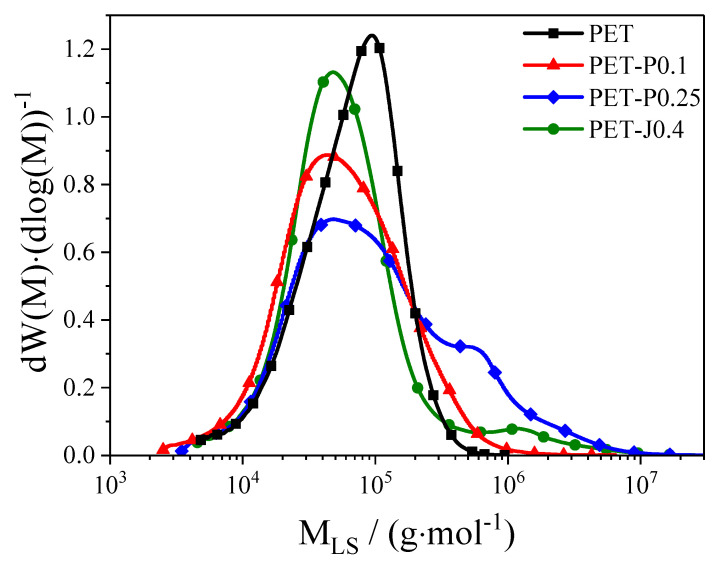
Logarithmic derivative of the cumulative molar mass distribution W (M) as a function of the absolute molar mass M_LS_ determined by light scattering for PET modified with PMDA or Joncryl [8,9]. The numbers of the PET samples describe the weight percentages of the modifier Joncryl (J) or PMDA (P).

**Figure 2:**
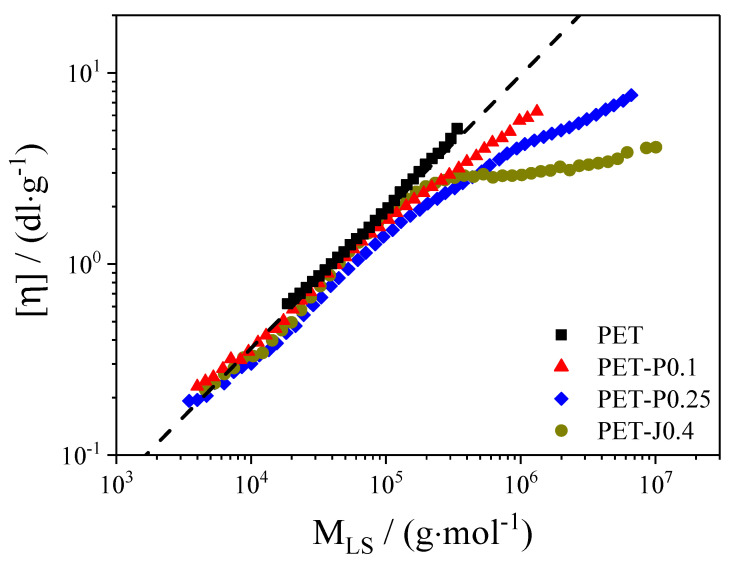
Intrinsic viscosity [η] as a function of the absolute molar mass M_LS_ determined by light scattering for PET modified with PMDA or Joncryl [8,9].

Figure 1 shows the molar mass distributions and Figure 2 the Mark–Houwink plot of the neat PET and the samples modified by reactive processing. The modified samples were prepared for a pre-study in a kneader using the same raw materials as for the film blowing experiments (cf. Experimental Section). The neat PET was analyzed as received from the supplier.

As follows from Figure 1, the common feature of both chain extenders is to broaden the molar mass distribution towards higher molar masses, and therefore to increase the weight average molar mass compared to the neat PET. Comparing the used concentrations in wt.%, the PMDA modifier shows a higher effectiveness and does not form any gel structures, as proven by solvent extraction. In contrast, the Joncryl modified sample of Figure 1 contains a gel content of 8 ± 2 wt.%, that was filtered out before performing the SEC measurements. Therefore, only the soluble part of the material is reflected by Figure 1 and Figure 2.

The linear molecular structure of the unmodified PET is confirmed by the straight line of the Mark–Houwink plot in Figure 2. For the modified samples, deviations from the straight line become obvious, especially in the high molar mass region, which indicate the existence of long-chain branches. The deviation is significantly more pronounced for the sample modified with Joncryl compared to the samples modified with PMDA, which points towards a more efficient branching structure [9]. A deeper discussion regarding the branching architecture and correlations with rheological properties can be found elsewhere [8,9,11].

### 3.2. Rheological Characterization

**Figure 3:**
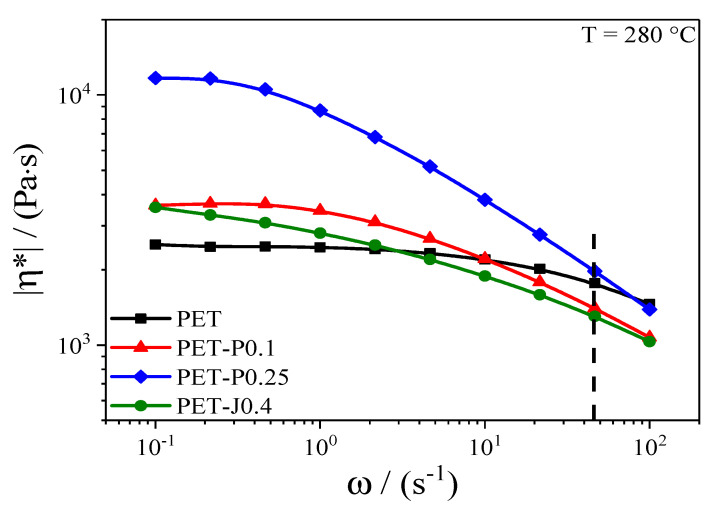
Magnitude of complex shear viscosity as a function of angular frequency for PET modified with PMDA or Joncryl. The vertical line represents the shear rate in the extruder during the film blowing experiments.

In Figure 3, the magnitude of the complex shear viscosity of the neat PET and the materials reactively processed in a twin-screw extruder is plotted as a function of the angular frequency at 280 °C. This temperature was used for the film blowing experiments. The neat PET shows a distinct Newtonian plateau at low frequencies and only at high frequencies a deviation towards lower shear viscosities. This weak shear thinning is a characteristic rheological behavior of a linear PET. The modification with the chain extenders leads to an increase of the viscosity in the low frequency range and a more pronounced shear thinning. The viscosity curves of all modified samples cross the viscosity curve of the neat PET within the frequency range chosen. The pronounced shear thinning is a result of the broader molar mass distribution compared to the unmodified PET and the introduced long-chain branches, as indicated in Figure 1 and Figure 2. In addition, the gel content in the Joncryl modified sample of 7 ± 1 wt.% influences the flow behavior. It is shown in [20] that adding 5% of a crosslinked linear low-density polyethylene (LLDPE) to a non-crosslinked LLDPE increases the viscosity in the low shear rate region, but has only a minor effect on the viscosity at high shear rates. No gel was detected for the PET, PET-P0.1, and PET-P0.25 samples.

**Figure 4:**
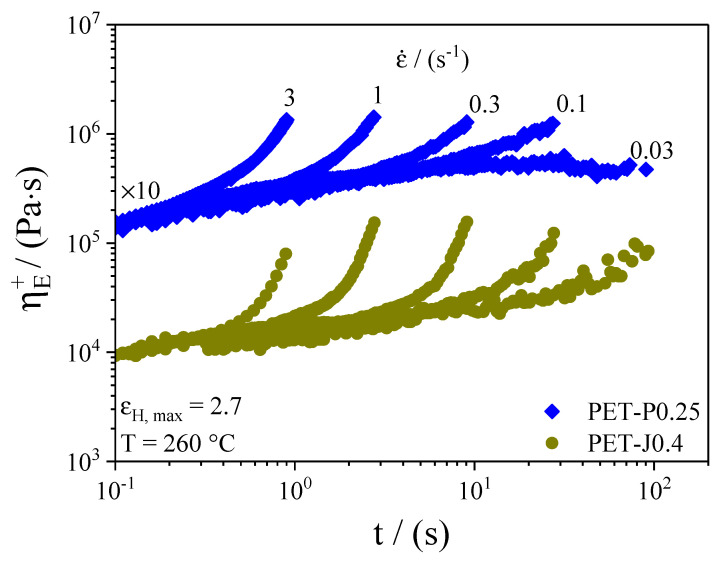
Time-dependent elongational viscosity at different elongational rates for PET-J0.4 and PET-P0.25. The PET-P0.25 curves are shifted by 10, as indicated in the figure.

The time-dependent elongational viscosity for different elongational rates at 260 °C is plotted for the samples PET-P0.25 and PET-J0.4 in Figure 4. The temperature has been lowered compared to the temperature used for the film blowing experiments because sagging of the samples may occur during measurements at high temperatures. Nevertheless, the melt strength at 260 °C of the samples PET and PET-P0.1 was still too low to measure the elongational viscosity. As can be seen in Figure 4, both samples show a distinct strain hardening, especially at high elongational rates. This is the well-known behavior for long-chain branched polymers [21]. Although the elongational viscosity of the neat PET could not be measured, it can be supposed that PET shows no strain hardening because the SEC measurements reveal no long-chain branches or high molar mass components.

**Figure 5:**
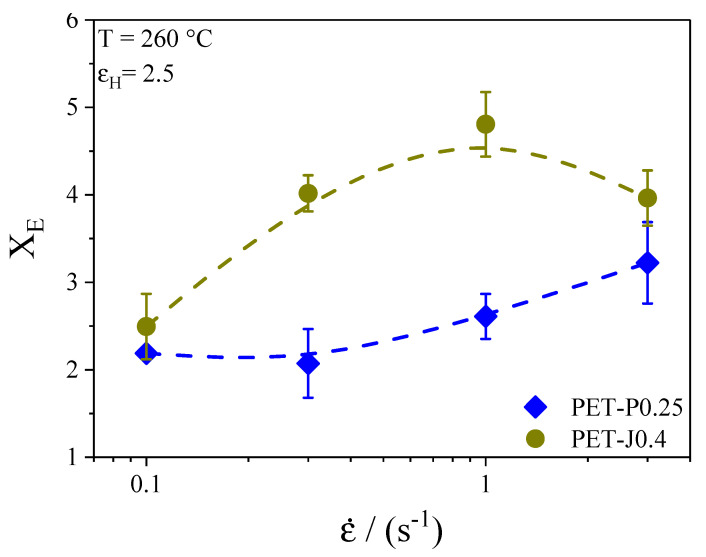
Strain hardening factor **X_E_** as a function of elongational rate $\dot{\varepsilon }$ at a Hencky strain of 2.5 for PET-J0.4 and PET-P0.25.

In order to compare the strain hardening of PET-J0.4 and PET-P0.25 from a quantitative point of view, the strain hardening factor **X_E_**_,_ defined as

(3) $$X_{E}=\frac{\eta_{E}^{+}\left(t,\dot{\varepsilon }\right)}{\eta_{E}^{+}\left(t\right)}$$

is calculated and plotted in Figure 5 as a function of the elongational rate at a constant Hencky strain of 2.5. For all elongational rates, **X_E_** of PET-J0.4 is higher compared to PET-P0.25. This is probably caused by a more efficient branching structure of the PET-J0.4 sample compared to the PET-P0.25 (cf. Figure 2) and by the gel content in PET-J0.4 which significantly increase the strain hardening [9,11,20].

**Figure 6:**
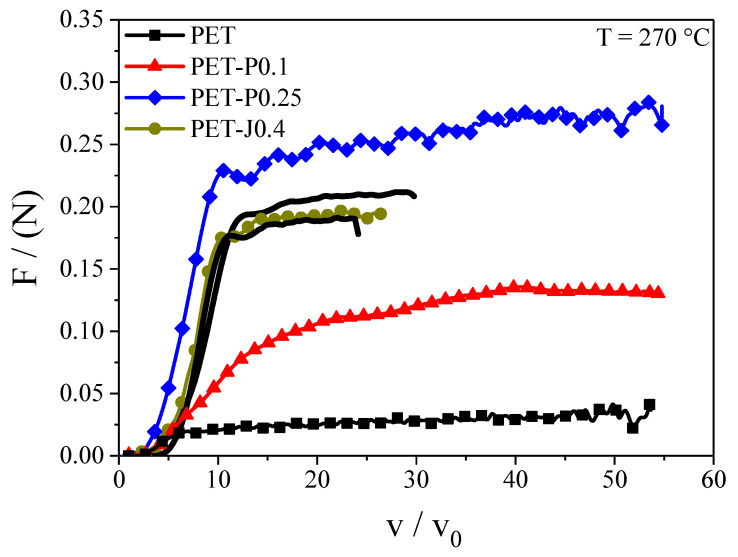
Tensile force F as a function of draw ratio v/v_0_ for PET modified with PMDA or Joncryl. The black lines indicate various measurements on the PET-J0.4 sample, to show the repeatability of the experiment.

A laboratory experiment to analyze the melt strength and extensibility of polymers is the Rheotens test. Figure 6 compares the results of the Rheotens test for all samples investigated. Due to the low melt strength of the neat PET, the temperature had to be reduced to 270 °C compared to the film blowing experiments. PET-P0.25 indicates the highest melt strength and a maximum draw ratio of 55, which is obviously not the physical limit of the material but represents the maximum velocity that can be obtained with the device used. The PET-P0.25 is followed by the PET-J0.4 and by PET-P0.1. The high melt strength of the modified samples is related to the special molecular characteristics, shown in Figure 1 and Figure 2. From literature [19,22], it is known that an increase in weight average molar mass, a broadening of the molar mass distribution, and long-chain branched structures raise the melt strength towards higher values. The neat PET displays the lowest melt strength due to the narrow molar mass distribution and the linear molecular structure. While the neat PET and the PET modified with PMDA can be drawn to the maximum draw ratio of 55, the extensibility of PET-J0.4 is reduced to approx. 26. This result agrees with investigations performed in [23], where PET modified with 0.3, 0.6, and 0.9 wt.% Joncryl was examined. The failure is probably due to the gel content. A reduced extensibility has also been reported for materials modified with high PMDA concentrations and obvious gel structures [13].

### 3.3. Film Blowing

#### 3.3.1. Extrusion Pressure

**Figure 7:**
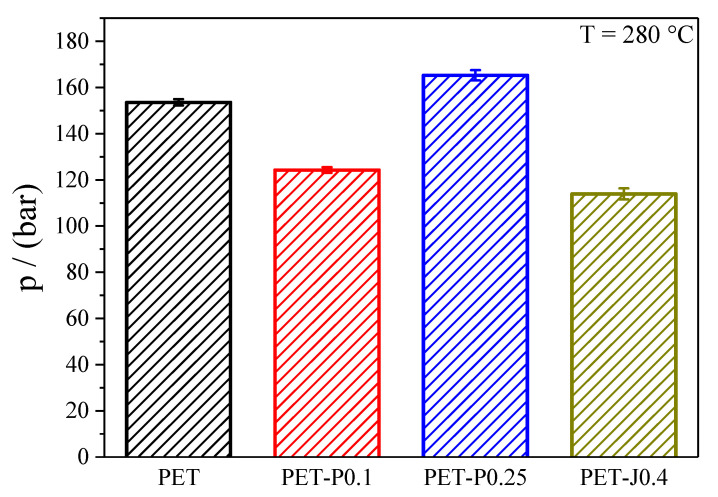
Extrusion pressure in the metering zone for PET modified with PMDA or Joncryl during the film blowing experiments.

The bar chart in Figure 7 demonstrates that the highest extrusion pressure during the film blowing experiments was observed for PET-P0.25, followed by PET, PET-P0.1, and PET-J.04. Remarkably, the reactively processed PET-P0.1 and PET-J0.4 samples show lower extrusion pressures compared to the neat PET. It is worthwhile discussing whether these differences in extrusion pressures are reflected by the viscosity data in Figure 3.

For this purpose, the shear rate $\dot{\gamma }$ in the single screw extruder was calculated according to

(4) $$\dot{\gamma }=\frac{N\cdot D_{s}\cdot \pi }{H_{s}}$$

where **N** is number of screw rotations per second, **D_s_** the screw diameter, and **H_s_** the screw flight depth [24]. Under the experimental conditions applied, a shear rate of 45 s^−1^ is obtained from Equation (4). For this shear rate, the viscosities of the four samples can be taken from Figure 3 making use of the Cox-Merz relationship. As a result, the pressure data in Figure 7 show the same ranking as the viscosity data. In addition, the relative differences between the viscosities are of a similar order of magnitude as those of the extrusion pressures (cf. Table 1). This result indicates the applicability of viscosity data for assessing the extrusion pressure in the single screw extruder used.

#### 3.3.2. Bubble Stability

An important feature for the performance of film blowing is the stability of the bubble. A stable bubble is important for films of high quality and, thus, it is a criterion determining the efficiency of a blowing process. Bubble stability is dependent on machine and processing parameters as well as on material properties. Investigations from the literature on widely used polyethylenes show that the rheological behavior in extension plays a decisive role (e.g., [14,25,26,27]). The topic addressed in this section relates to whether these results can be transferred to modified PET.

**Figure 8:**
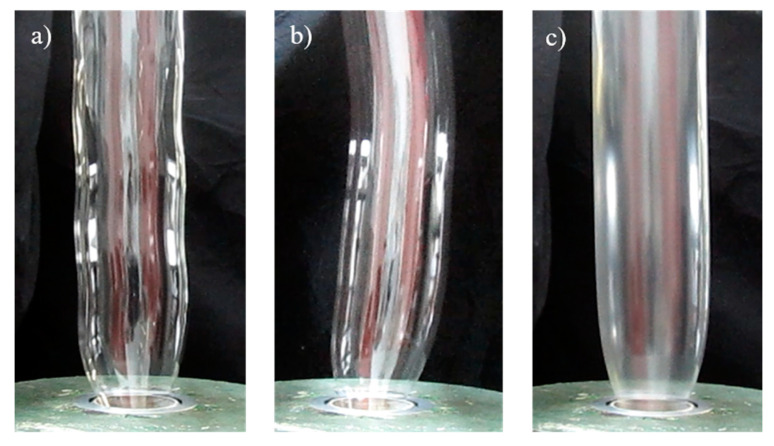
Image of (**a**) an axisymmetric periodic variation of the bubble diameter, (**b**) a helical motion of the bubble, and (**c**) a stable bubble. Images (**a**) and (**b**) show the neat PET and (**c**) the PET-P0.25 sample. The processing conditions are listed in Section 2.4.

In general, three kinds of instabilities are distinguished. The first one is described by an axisymmetric periodic variation of the bubble diameter, the other by a helical motion of the bubble and a third by a variation of the frost line. In addition, superpositions of these instabilities can be observed. Figure 8a,b demonstrates the first two instabilities occurring for an unmodified PET processed with the laboratory equipment described in Section 2.4.

Figure 8c distinctly demonstrates that a stable bubble contour could be obtained for the PET modified with PMDA. Because a quantitative criterion for bubble stability is obviously not available, the assessment is visually performed, and is therefore of a qualitative nature. Nevertheless, a distinct ranking of the bubble stability of different products could be experimentally found in the order

PET-P0.25 > PET-J0.4 > PET-P0.1 > PET.

The performance of PET-J0.4 exhibited a peculiarity insofar as a breakdown of the bubble occurred from time to time during processing at high **TUR**. This may be due to the gels found within this Joncryl-modified sample giving rise to mechanical failure.

In the literature and particularly convincingly described for various polyethylenes in [27], the bubble stability is related to the melt strength of a sample determined in a Rheotens test. Such measurements on PET and three modified samples are presented in Figure 6. They show distinct differences in the height of the drawing forces from the lowest value for the unmodified PET to the highest for PET-P0.25. The ranking corresponds very well with the bubble stability listed above. Even the observation of the processing performance of PET-J0.4 is reflected by the Rheotens experiments that show an earlier failure than for the three other products, which may be explained by the gel content.

These results are in line with investigations performed on Joncryl modified PLA [15,16] and on PMDA modified PET [13], where an improved bubble stability has been found compared to the unmodified polymer. In addition, it is reported in [13] that, at high PMDA concentrations (0.75 wt.%), a gel is formed that reduces the drawing capability, as observed in the Rheotens experiment and during film blowing.

The results demonstrate two insights considering the significance of even qualitative rheological experiments in extension like Rheotens tests for processing operations as film blowing. First, they allow an at least comparative assessment of the bubble stability of various polymeric materials, and second, the findings may be used for product development.

#### 3.3.3. Film Thickness Uniformity

**Figure 9:**
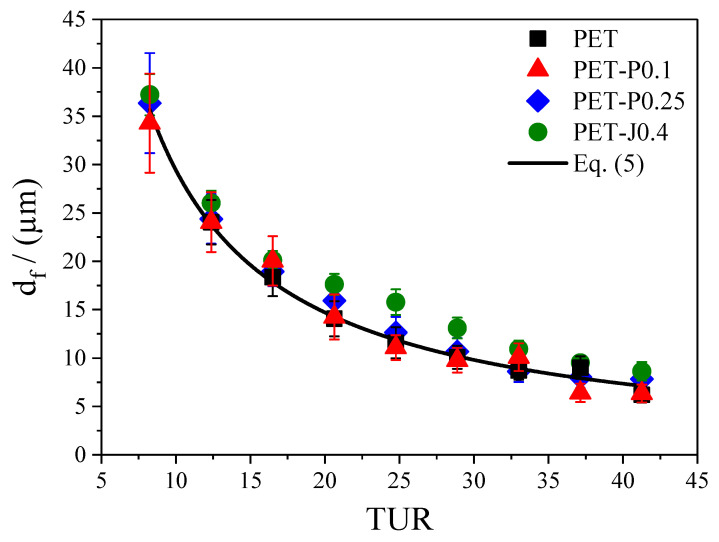
Measured film thickness d_f_ as a function of take-up ratio **TUR** for PET modified with PMDA or Joncryl in comparison to the data calculated according to Equation (5).

Only a few studies can be found in the literature dealing with the thickness uniformity of blown films [14,28,29]. Moreover, none of them used PET or reactively processed PET. To evaluate the film thickness uniformity, the following procedure was applied. The thickness at each **TUR** was measured at 20 positions with a distance of 1 cm in take-up and blow-up directions. This was repeated five times for each **TUR**. The average values and standard deviations for all films are plotted in Figure 9. As can be seen, the average film thickness d_f_ decreases with **TUR** down to approx. 7 µm and according to [30], can be well predicted by

(5) $$d_{f}=\frac{{\left(d_{1}/2\right)}^{2}-{\left(d_{0}/2\right)}^{2}}{2d_{1}}\frac{\rho_{M}}{\rho_{S}}\frac{1}{BUR\cdot TUR}$$

where **d**_0_ is the inner and **d**_1_ the outer diameter of the ring die, $\rho_{M}$ the melt density, and $\rho_{S}$ the density of the film.

With the average thickness $d_{f}$ and the standard deviation **σ**, the relative standard deviation **σ_r_**, also called inhomogeneity index or non-uniformity index [14,27,28,29], can be calculated accordingly:

(6) $$\sigma_{r}=\frac{\sigma }{d_{f}}\cdot 100\%$$

**Figure 10:**
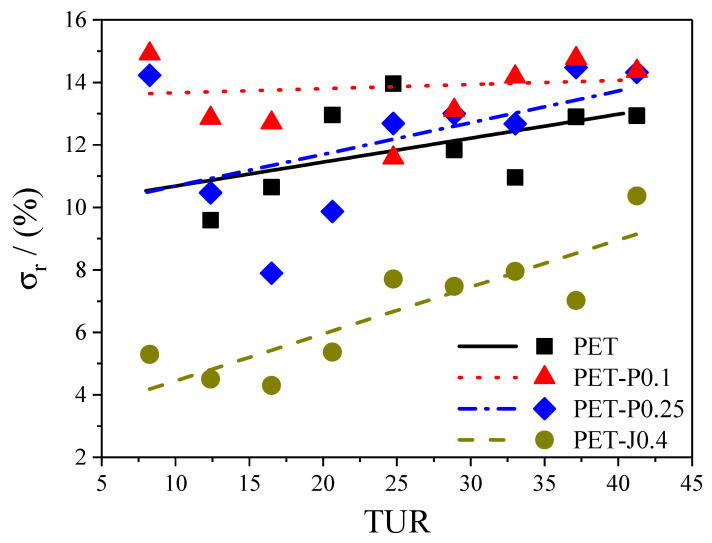
Non-uniformity index **σ_R_** as a function of take-up ratio **TUR** for PET modified with PMDA or Joncryl. The lines are a guide to the eye.

This value is plotted as a function of **TUR** in Figure 10.

In general, for all materials investigated (except for PET-P0.1), the non-uniformity index increases distinctly with growing **TUR**. This observation is in line with investigations on various polyethylenes [14,27,28]. Meanwhile, within the accuracy of the measurement, hardly no differences regarding film thickness uniformity can be detected between the neat PET and the samples with various PMDA contents, and the Joncryl modified material shows a significant improvement at all **TUR**. From this result, it can be concluded that the bubble stability and the height of the melt strength, measured by the Rheotens experiment constitute no indication of a better thickness uniformity (cf. Section 3.3.3). This is in agreement with other studies [14,27,28]. The improved thickness uniformity of PET-J0.4 compared to the neat PET can be ascribed to the pronounced strain hardening behavior (cf. Figure 4 and Figure 5), which results from the branching structure (cf. Figure 2) and the gel content [8,9]. This is in line with [14], where it is convincingly shown that strain hardening is required to obtain an improved thickness uniformity. Even though the elongational viscosity of the neat PET cannot be measured due to sagging of the specimen in the rheometer, it can be expected from the molecular characteristics in Figure 1 and Figure 2 that it shows no strain hardening due to the linear molecule structure and the absence of a high molar mass component [21]. These differences in molecular structure and rheological behavior explain the better uniformity of the PET-J0.4 sample compared to the neat PET.

Taking these results into account, it is surprising that the PET samples modified with PMDA show no improved thickness uniformity. Both samples reveal long-chain branched structures, proven by the Mark–Houwink plot (Figure 2), that effect a strain hardening behavior of the PET-P0.25 sample (cf. Figure 4 and Figure 5). The PET-P0.1 cannot be measured by elongational rheometry, however it can be expected that the long-chain branched structures lead to a strain hardening, but probably not as pronounced as for the PET-P0.25 material. In order to compare PET-P0.25 and PET-J0.4, the elongational viscosity and the strain hardening factor at $\varepsilon_{H}$ = 2.5 and $\dot{\varepsilon }$ = 0.3 s^−1^ for both samples are listed in Table 1. A Hencky strain of 2.5 and an elongational rate of 0.3 s^−1^ can be seen as representative values for the film blowing application [31]. Comparing the elongational viscosity between PET-P0.25 and PET-J0.4 indicates that they are of a very similar order of magnitude (cf. Table 1). This gives some indication that the absolute value of the elongational viscosity is not the parameter that correlates with the thickness uniformity. However, the strain hardening factor of the PET-J0.4 sample is significantly higher compared to the PET-P0.25 sample, especially at 0.3 s^−1^, but also for lower and higher strain rates (cf. Figure 5). From this result, it can be concluded that an improved thickness uniformity is related to samples showing a pronounced strain hardening and that low values of the strain hardening factor might not be sufficient to significantly affect the uniformity of blown films.

## 4. Conclusions

From a processing point of view, a suitable polymer for film blowing applications should provide a low extrusion pressure, a good bubble stability, a good drawability, and a small film thickness variation in take-up and blow-up directions. Reactive processing with chain extenders like PMDA or Joncryl^®^ ADR 4368 is an elegant way to change the rheological behavior of commercially available PET to make it more suitable for the film blowing process. In particular, it has been shown that adding 0.4 wt.% Joncryl leads to a reduced extrusion pressure due to pronounced shear thinning, an improved bubble stability, and a better film thickness uniformity compared to the neat PET (cf. Table 1). These improvements can be ascribed to an increase in weight average molar mass, a broadening of the molar mass distribution, and the formation of long-chain branches. However, gels occur that lead to a reduced drawability in the Rheotens test and an occasional breakdown of the bubble in the laboratory experiment at high **TUR**. The PMDA modified samples show good drawability and no gels, leading to a superior bubble stability at high PMDA concentration. The disadvantage of PMDA is a less efficient branching structure, resulting in only a small improvement of the thickness uniformity compared to the neat PET.

Taking these findings into account, a PET material suitable for film blowing applications might be created by a two-step reactive process: (1) extrusion with PMDA in order to improve bubble stability by increasing weight average molar mass, broadening of molar mass distribution, and generation of branched structures, and (2) adding Joncryl in a later stage of the reactive processing in order to introduce an efficient branching structure at low gel contents, to raise the strain hardening and to improve the thickness uniformity. Some hints towards such a two-step reactive process can be found in the patent literature [32] and in a recent study performed with PLA and PMDA and Joncryl as chain extenders [33].

## Figures and Tables

**Table 1:** Summary of the rheological properties and correlation with characteristic film blowing parameters. The applied temperatures are listed in the Experimental Section.

	PET	PET-P0.1	PET-P0.25	PET-J0.4
Complex Viscosity ^1^ at 45 s^−1^/Pas	1780 (100)	1410 (79)	1990 (112)	1310 (74)
Extrusion Pressure ^1^ at 45 s^−1^/bar	154 (100)	124 (81)	165 (107)	114 (74)
Melt Strength/N	0.03	0.13	0.27	0.19
Extensibility (v/v_0_)	55 ^2^	55 ^2^	55 ^2^	26
$\eta_{E}^{+}$ at $\varepsilon_{H\;}$= 2.5, $\dot{\varepsilon }\;$= 0.3 s^−1^/kPas	n.m. ^3^	n.m. ^3^	110	101
X_E_ at $\varepsilon_{H}\;$= 2.5, $\dot{\varepsilon \;}$= 0.3 s^−1^	n.m. ^3^	n.m. ^3^	2.1	4.0
Bubble Stability ^4^		_+_	_++_	_+_^5^
Film Thickness Uniformity ^4^		O	O	_++_

^1^ Numbers in brackets refer to the difference in % compared to PET. ^2^ Technical limit of the Rheotens for the settings used. ^3^ Not measurable (cf. Section 3.2). ^4^ O equal to PET, + better than PET, ++ much better than PET. ^5^ Breakdown at high **TUR** possible.
